# Mechanisms of improved survival from intensive followup in colorectal cancer: a hypothesis

**DOI:** 10.1038/sj.bjc.6602369

**Published:** 2005-02-01

**Authors:** A G Renehan, M Egger, M P Saunders, S T O'Dwyer

**Affiliations:** 1 Department of Surgery, Christie Hospital NHS Trust, Wilmslow Road, Manchester M20 4BX, UK; 2 Department of Social and Preventive Medicine, University of Beme, Switzerland; 3 Department of Clinical Oncology, Christie Hospital NHS Trust, Manchester, UK

**Keywords:** followup, colorectal cancer, recurrences, salvage surgery, meta-analysis

## Abstract

A meta-analysis of six randomised trials demonstrated that intensive followup in colorectal cancer was associated with an absolute reduction in all-cause 5-year mortality of 10% (95% confidence interval (CI): 4–16) – however, only two percent (95% CI: 0–5) was attributable to cure from salvage re-operations. We postulate that other factors, such as increased psychological well-being and/or altered lifestyle, and/or improved treatment of coincidental disease may contribute to the remaining lives saved, and form important future research questions.

Approximately two-thirds of patients presenting with colorectal cancer undergo resection with curative intent, and most subsequently enter protocols for long-term followup (Kievit, 2002). The rationale for surveillance is three-fold: psychological support, facilitation of audit, and an opportunity for the early detection and treatment of recurrent disease, with potential improvement in survival. Recently, the authors (Renehan *et al*, 2002b) reported a meta-analysis of five randomised trials and demonstrated a significant improvement in all-cause 5-year mortality in patients followed intensively. A Cochrane review (Jeffery *et al*, 2002) independently found similar results, and, subsequently, a sixth randomised trial reported additional results supporting these conclusions (Secco *et al*, 2002). These data offer the first direct evidence that intensive followup improves survival, but fall short of evaluating the mechanisms underlying the observed survival benefit. This study updates and extends our previous meta-analysis to explore the survival mechanisms associated with intensive followup.

## MATERIALS AND METHODS

The search strategy, inclusion and exclusion criteria, data extraction, and study quality assessment have been published elsewhere (Renehan *et al*, 2002b, 2004), with further details at www.christie.man.ac.uk/profinf o/departments/surgery/default. htm. The key features were:

- Updated search strategy (to December 2003) using Cochrane methodology.
- Inclusion criteria were: randomised controlled trial; patients with colorectal cancer treated surgically with curative intent; randomisation at or shortly after surgery, and availability of 5-year survival data.
- Data were extracted independently by two investigators (AGR, MPS).
- Important components of methodological quality, namely adequacy of concealment of patients' allocation to treatment groups, double-blinding, and withdrawals, were assessed.

## STATISTICAL ANALYSIS

Meta-analyses were performed at two levels. First, comparisons of events (e.g. all-cause deaths) for intensive *vs* conventional followup were performed and pooled estimates expressed as risk ratios (RR) and 95% confidence intervals (95% CIs). Second, estimates of the proportion of overall lives gained, and lives gained through to salvage re-operation, were calculated using absolute risk differences (and 95% CIs) (Deeks and Altman, 2001). The difference between these estimates was taken as an estimate of the gain in lives attributable to nonsalvage-related factors. Tests for heterogeneity were performed, and, if significant, sources of heterogeneity were explored. Random-effect methods were used throughout and all tests were performed using STATA™ version 7.0 (Stata corporation, College Station, TX, USA).

## RESULTS

### Baseline characteristics

There were six randomised controlled trials (Makela *et al*, 1995; Ohlsson *et al*, 1995; Kjeldsen *et al*, 1997; Pietra *et al*, 1998; Schoemaker *et al*, 1998; Secco *et al*, 2002) comprising 1679 participants, 858 assigned to intensive and 821 assigned to conventional followup (webappendix1). The surveillance tests, and the frequency of their use, varied considerably (webappendix2).

In general, trial methodology was moderate. Two trials (Pietra *et al*, 1998; Schoemaker *et al*, 1998) reported allocation by open cards or random number tables. Randomisation was stratified by site and Dukes' stage in two trials (Kjeldsen *et al*, 1997; Schoemaker *et al*, 1998). Specifically, with relation to this analysis, blinding of clinicians and/or assessors about interpretation of recurrence detection and/or decisions about subsequent treatments were not mentioned, except for one trial (Schoemaker *et al*, 1998). However, followup rates among survivors were generally good.

### Outcomes

There were 268 (31%) deaths in patients followed intensively compared with 328 (40%) for those followed by conventional regimens, giving a pooled RR estimate of 0.76 (95% CI: 0.67–0.86) (Table 1). The recurrence rates for all sites were similar in both arms (36 *vs* 37%), but re-operation rates favoured those intensively followed (9 *vs* 6%: RR=2.12, *95*% CI: 1.43–3.15). For both arms, the proportions successfully salvaged were low – 4 and 2%, respectively.

**Figure 1:**
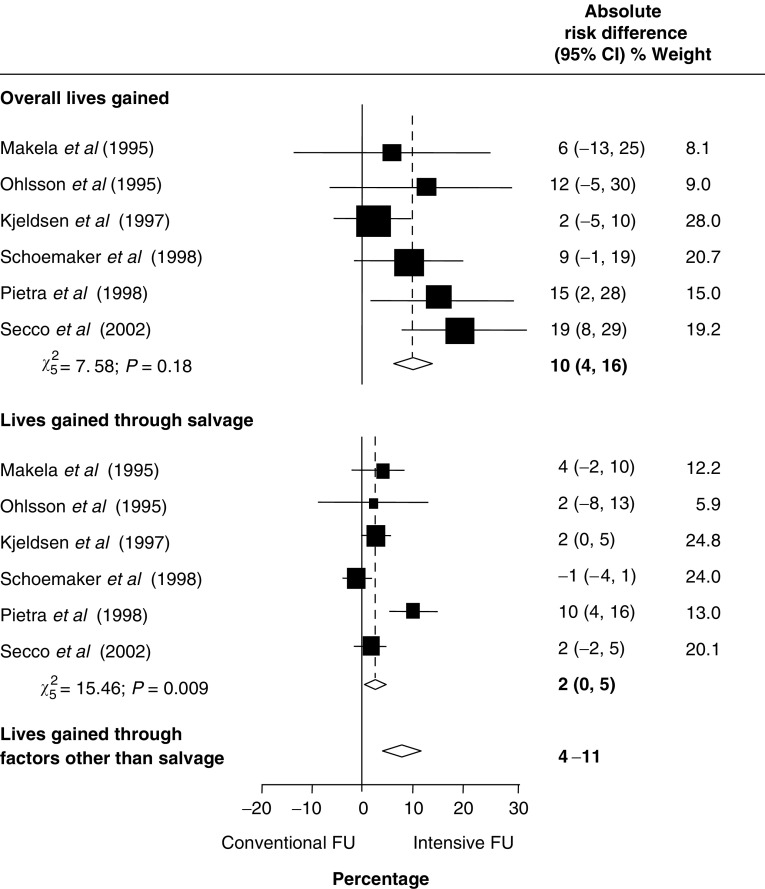
Absolute risk differences for overall lives gained, lives gained through salvage and through factors other than salvage: random-effects method. CI: confidence interval. FU: followup.

The estimate of absolute risk difference for overall lives gained from intensive followup was 10% (95% CI: 4–16), but only 2% (95% CI: 0–5) was attributable to cure from salvage re-operation (Figure 1). The difference (4–11%) suggests that factors other than salvage may contribute to survival from intensive followup. There was significant statistical heterogeneity (*P*=0.009), which, after exploration, was mainly due to the Pietra *et al* (1998) trial, a study which had high re-operation rates among patients intensively followed (20 *vs* 6%). After excluding this study, the absolute risk difference for all lives gained was 9% (95% CI: 2–16; *χ*_4_^2^=6.70, *P*=0.15); that for lives gained through salvage was 1% (95% CI: 0–3; *χ*_4_^2^=6.38, *P*=0.17).

## DISCUSSION

The findings of this study add to earlier meta-analyses (Jeffery *et al*, 2002; Renehan *et al*, 2002b; Figueredo *et al*, 2003), indicating improved survival with intensive followup after curative resection for colorectal cancer. In exploring the mechanisms of this benefit, the present study shows that salvage surgery offers a ‘second chance’ of cure in a small number of cases (up to 5%), but that an additional 4–11% gain in survival may be attributable to other factors.

There are a number of potential limitations to this interpretation. Blinding of outcome assessment was generally poorly reported, such that factors determining the decision to re-operate may be biased. On the other hand, in one trial (Secco *et al*, 2002), a substantial proportion of control patients had ‘asymptomatic’ recurrences, suggesting that the intervention (i.e. intensive followup) may have contaminated the control arm. Other limitations included the clinical heterogeneity of followup regimens among the trials considered, the lack of quality of life data, and that most of the included trials were carried out over a decade age and thus may not represent contemporary oncological practice. Additionally, even within a meta-analysis of six trials, the number of recurrences treated and cured (disease-free) at the end of the study period was small, and, consequently, estimates of risk differences for disease-free status post-salvage surgery were associated with wide CIs.

An advantage of the present analysis is that it brings together a number of randomised studies, which individually were too small to address followup mechanisms. Only studies where randomisation occurred at the time of initial surgery were included, and survival was calculated at 5 years after initial treatment, hence circumventing potential lead and lag biases. The reported absolute overall survival benefit of 10% is similar to that estimated independently by others (Ohisson and Paisson, 2003), while the figure of 2% of lives saved through salvage alone is close to the 2.4% estimated using a recurrence–salvage–outcome model (Kievit, 2002). This apparent discrepancy emphasises the importance of determining the effectiveness of colorectal cancer followup based on all-cause mortality, as judging effectiveness simply based on the number of asymptomatic recurrences detected and salvaged underestimates the true potential impact of intensive followup.

From the observations in this study, we hypothesise that factors other than salvage treatment may contribute to improved survival in colorectal cancer followup. The following may be relevant: (i) increased psychosocial support and well-being; (ii) altered dietary and lifestyle factors and (iii) improved treatment of coincidental disease. For the first postulate, there is evidence that psychological therapies improve outcome in some cancer types (Newell *et al*, 2002), and at least one randomised trial (Kuchler *et al*, 1999) has shown improved survival using psychological support techniques among patients with gastrointestinal malignancies, of which one-third were colorectal cancers. Health-related quality of life was determined in one trial included in the meta-analysis (Kjeldsen *et al*, 1999), which showed a small benefit associated with intensive followup. Moreover, based on observations in breast cancer patients, there is a perception that intensive followup is associated with increased anxiety, but this has not been demonstrated in studies of followup in patients with colorectal cancer (Stiggelbout *et al*, 1997; Kjeldsen *et al*, 1999). Indeed, in the Danish randomised trial (Kjeldsen *et al*, 1999), the proportions of patients who were ‘never nervous’ favoured the intensively followed group when evaluated both before and after their planned clinical visit. In support of the second postulate, persistence of adverse lifestyle factors, such as obesity (Calle *et al*, 2003) and continued smoking (Goodman *et al*, 1990) after treatment of a primary malignancy, are associated with decreased survival for certain cancer types. Furthermore, cancer survivors tend to make substantial changes to their diet and lifestyle (Patterson *et al*, 2003), though it is unclear whether these adjustments are selfmotivated or a direct consequence of health-care interventions. Changes in patient behaviour due to feeling ‘observed’ are recognised – known as *Hawthorne* effects (Braunholtz *et al*, 2001), but whether or not frequent clinical assessments during colorectal cancer followup is relevant in this regard merits further study. The third postulate is an example of a ‘care effect’ within a trial (Braunholtz *et al*, 2001), and it is conceivable that followup *per se* may be a sophisticated mechanism of ensuring regular medical contact and may benefit patients irrespective of their cancer.

If the hypotheses developed from this study are true, the implications for future health resources and research are considerable. For decades, the majority of colorectal cancer followup was performed by clinicians within surgical and/or oncology clinics. Alternatives to this are emerging, and include colorectal nurse specialists working in parallel with colorectal cancer specialists using protocol-driven protocols (Renehan *et al*, 2002a). In this setting, there may be a role for additional allied disciplines such as cancer nutritionists and psychologists. These possibilities now form important future research questions.

## Figures and Tables

**Table 1:** Summary estimates: random-effects methods

	**Intensive FU**	**Control FU**	**Risk ratio (95% CIs)**	**Test for heterogeneity**
All-cause deaths	268/858 (31)	328/821 (40)	0.76 (0.67, 0.86)	*χ*_5_^2^=5.51; *P*=0.36
All site recurrences	313/858 (36)	307/821 (37)	0.94 (0.83, 1.06)	*χ*_5_^2^=1.55; *P*=0.91
Re-operation rates	79/858 (9)	33/821 (4)	2.12 (1.43, 3.15)	*χ*_5_^2^=4.33; *P*=0.50
Salvage cure rates	34/858 (4)	33/821 (2)	2.18 (0.86, 5.49)	*χ*_5_^2^=7.76; *P*=0.17

Unless otherwise stated, values in parentheses are percentages.

CI: confidence interval. FU: followup.
